# Some of the Unsuspected Effects of the Bromides*Read before the Richmond Academy of Medicine and Surgery, August 25, 1896.

**Published:** 1896-10

**Authors:** J. Allison Hodges

**Affiliations:** Professor of Nervous and Mental Diseases, University College of Medicine, Richmond, Va.


### SOME OF THE UNSUSPECTED EFFECTS OF THE BROMIDES.*

BY J. ALLISON HODGES, M.D.,

Professor of Nervous and Mental Diseases, University College of Medicine, Richmond, Va.

Regarding the effects of the bromides on the system in large or continued doses, not a little difference of opinion has existed. Ordinarily, these salts are considered by the profession as harmless in their constitutional effects, and the limitation of the dose, or the duration of their exhibition, is gauged only by the idiosyncrasies of the patient, or the personally conceived demands of his own nervous condition. The usual carelessness of the physician in this respect is as reprehensible and dangerous, in my opinion, as the recklessness with which the patient becomes accustomed consequently to use these drugs for every supposed nervous manifestation. In small and interrupted dosage, the bromides are calmative and effective remedies, and in the great majority of patients, act as a moderate sedative to the nervous functions generally, without any obvious ill effects.

This fact often causes their indiscriminate and continuous use, and subsequently there supervene certain physiological effects which are attributed in many instances to the disease under treatment, as one of its manifestations, instead of to the agents which are being used to combat it. This is manifestly true in those cases where large doses have been given, and I think in those cases as well where small doses have been continued for a long period, the ultimate constitutional effects of course being the same. If these

*Read before the Richmond Academy of Medicine and Surgery, August 25, 1896.

latent and commonly overlooked effects, which I shall mention, were always as patent as the bromism, which eventually comes on after long-continued employ of these salts, thus showing resentment at their farther ingestion, it would be needless to direct attention to them.

But such is not the case. On the other hand, many of the physiological effects are masked and obscured by other temporarily existing conditions. Originally, and even as late as 1870, the bromides, and especially the bromide of potassium, was considered an alterative and deobstruent, ([1]) and analogous to the preparations of iodine. It was proved experimentally, however, a few years later, that they were nervous sedatives, and, specifically, cerebral sedatives, and no clinical fact is more conspicuously verified than that of their efficacy in these and allied pathological conditions. Their ultimate effect on the system, however, is yet a *quæstio vexata*; some authorities maintaining that since some patients have taken them largely and continued them for a long time without injurious result, or, indeed, any very striking result of any kind, that they are consequently harmless; while other experimenters, on the contrary, have found them, even in moderate doses, to exercise powerful influences on the constitution, and in larger doses, or too long continued, to produce peculiar poisonous effects.

Most physicians, I believe, are prone to overlook and disregard the true physiological effects of these medicaments, because they attribute the induced symptoms to the primary neurotic condition of the patient, and in the end are often surprised to find an unaccountable depression of vitality, both physical and mental, that far outweighs the evident control of the nervous symptoms that had been obtained by this treatment.

I am free to admit that such was formerly my experience, having been taught the innocuousness of the bromides, until it was forcibly impressed upon me some years ago by several cases that staggered my credulity, and more recently, when that recognized authority, Dr. Weir Mitchell, by his experience of their baleful effects, has enforced the lesson upon me.

As to their toxic effects, the bromide of potassium is recognized as the most powerful, and the bromide of sodium as the least toxic. There is a general correspondence, physiologically, in the action of all the bromides, except that of ammonium, due, doubtless, to its base, and they are all very diffusible, which accounts in some measure for the no more frequent ill effects observed, for it is certain that when administered in large doses no inconsiderable portion escapes absorption, being easily detected in the intestinal mucus, etc. Most persons take the usual doses with no perceptible disadvantages other than acne, roughened skin, etc., but the prolonged administration develops in all cases that condition of chronic poisoning known as bromism, which differs from the effects of a few medicinal doses in the extent and intensity, but not in the character, of the symptoms.

If the bromides are to be continued for any indefinite period, it is difficult to determine what is a safe dose, for it is clinically substantiated by Voison (2) that 60 grains, repeated for from three to six times, at intervals, with some persons will occasion slight nausea and diarrhea, while Bartholow (3) has ascertained that 100 grains for one dose will lower the temperature in a healthy adult from one-fifth to one-half a degree, the respirations from two to five, and the pulse from ten to twenty beats per minute.

It is some of the unusual, or, at least, not usually suspected, effects of the bromides that I desire to consider in this article, symptoms which, in their milder forms, must be familiar to all practitioners, but which sometimes, I think, are attributed to a mistaken parentage, to the disease and not to the remedy. Among these commonly unsuspected effects may be mentioned the following:

1. Increased irritability of temperament.

It is a notable fact that most patients who have undergone the bromide treatment for any considerable length of time, for nervousness, insomnia, etc., develop an irritable, peevish, and querulous disposition, disproportionate to the exciting cause. This is particularly noticeable in epileptics, and is often assigned as one of the manifestations of the disease itself. This, I am confident, is a mistaken assumption, for, as a clinical fact, attested in many of these cases, in my experience, the temporary suspension or withdrawal of the treatment at once moderates and often completely suspends the irritable tendency. In the case of epileptics, also, it is true that this irritability is increased, even though the convulsions be lessened in their severity. Many nurses, even, will bear testimony to this fact.

2. A depression of spirits, with a tendency to moderate melancholia.

There are some neurotic cases in which moderate doses of the bromides occasion despondency in the individual, which, at times, amounts almost to a melancholia, though this is rare.

In June of last year, I was consulted by a lady of a neighboring city, who came to me with the history of grave melancholy. She was intensely depressed, and in constant fear of "losing her mind." She was about 45 years old, and a great sufferer from insomnia. Upon examination, no physical defects were discoverable, and, but for a slightly anemic condition, she was in fair physical health. Inquiry elicited the fact that, one year before, when suffering from pelvic pain, a mixture of bromides, containing about 80 grains a day, had been prescribed by her physician; and, fearful of a return of the trouble, and to insure sleep, she had almost constantly maintained this treatment ever since. At my solicitation the treatment was partially suspended, and in two weeks there was marked improvement. At the end of a month, upon the entire cessation of the bromides, there was almost complete restoration to health. Later, with the aid of bitter tonics, she was restored to her normal condition, and up to this date is in perfect health, and has had no return of melancholia.

3. Impairment of memory and also of contractility of muscles.

In nearly all cases, where large or continued doses of these drugs are taken, there is, to a greater or less extent, an impairment of memory. Weakness of mind, without perversion of intellect, is a very constant result of the use of large doses. Failing memory, especially in epileptics, who, according to the general routine of practice, take the greatest quantity of bromides, is almost invariably assigned to the disease as the causative factor, but my experience demonstrates that the bromides themselves are, for the most part, responsible for this condition. Dr. Mitchell ([4]) reports the case of two children, ages 10 and 13, respectively, suffering with mild epilepsy, who, owing to a mistake of the nurse, were given 100 grains a day of lithium bromide for a few days. Memory was impaired in both. One forgot the letters of the alphabet, and the other had some curious confusion as to the time of events, misdating them. This state of altered memory, in both cases, passed away in two days after the bromides were withdrawn. These cases also illustrate that motility is impaired by the bromides, for the same author relates that the physical weakness in both was very marked, and that while both children could stand, neither could walk. The bromides evidently possess the power to destroy or impair the irritability of the motor and sensory nerves, and the contractility of muscle; and Dr. Rudisch ([5]) has pointed out that the paretic symptoms first manifest themselves in a tendency to ptosis, and, later, an increasing feebleness of all the limbs, amounting, in some instances, almost to a hemiplegic condition. While it is not often observed, still several cases are reported where large doses, given by mistake, or reckless lay use of the drug, have induced a complete relaxation of the sphincters. A prompt withdrawal of the drug has always restored the functions to their usual offices.

4. Irritant to the gastric mucous membrane.

In small doses, well diluted, the bromides are generally acceptable to the stomach, and by some believed to be positively anesthetic to the mucous membrane. In large doses, or in continued administration, however, they are distinctively irritant. This point is clinically well established as regards large doses, but it may not be so evident in the case of continued moderate doses, which in effect, however, produce the same results.

Several years ago a lady consulted me for diarrhea, and thinking it was of nervous origin, I administered the bromide of sodium in 30-grain doses, thrice daily, in conjunction with other treatment. Upon her return, several days later, I found her condition not only not improved, but worse. She informed me that, not having been benefited by my prescription, she had also taken larger doses of the bromides that she had been accustomed to taking when she felt "out of sorts," along with the medicine I had given her. Learning that it had been her habit to take several doses a day of bromide of potassium for some months, my suspicions were aroused that that drug, intensified in its irritant effects by the 90 grains daily I had ordered, was to blame for her non-improvement. A suspension of treatment, with rest and boiled milk diet, speedily effected a cure.

5. Bromides act as an antaphrodisiac.

Some authors affirm that, if given in large doses, they diminish and, at length, if long continued, entirely subdue the venereal appetite. M. Puchi ([6]) insists that, under these conditions, the patient will fall into a condition of impotence, which sometimes outlasts the use of the medicine several days. My experience, however, has not confirmed the antaphrodisiac properties of these drugs.

6. They disturb the circulation.

Very obvious effects are produced on the action of the heart when large doses are administered. Bartholow ([7]) says that the number of the cardiac pulsations is not only reduced, but their force is diminished, and the tension of the arterial system is lowered. Dr. Da Costa indorses this statement and inveighs against the use of the bromides in those persons having weak hearts, "especially the form of cardiac weakness designated as chronic cardiac asthenia."

7. Extraordinary susceptibility to toxic effects in cases of cerebral lesions, etc.

Several recognized authorities speak of the sudden bromic toxication liable to occur in persons who have suffered cerebral lesions from trauma, or who have had emboli or necrotic processes around clots. Dr. Mitchell ([8]) reports a case of rheumatism in a young man who had an embolus in the right middle cerebral artery, and whose condition presented increasing evidences of destructive irritative changes in the brain. To control this he gave 60 grains of potassium bromide daily for three days. Acute mania was the result, and was not relieved until he ceased the treatment. Surgically, this is an important fact, and should be heeded.

8. Tendency to give rise to homicidal and suicidal impulses.

MM. Rames and Huette ([9]) were the first that I can learn to chronicle any form of intoxication or mental derangement from the employment of the bromides in large doses. Hammond ([10]) and others have also recorded cases of mental derangements with hallucinations of a melancholic character, but Escheverria was the first to call attention to the tendency to homicidal and suicidal impulses. The first case that ever caused me to look with suspicion upon the bromides as capable of producing sudden toxic effects was one of this nature. In 1890, I was called to see a patient, female, aged 65, and found her to be a nervous, miserable creature, emaciated, feeble, and hysterical. I administered the bromide of sodium in 60-grain doses daily, and there was some improvement in the nervous symptoms. Subsequently, on account of expense, I ordered the salt in crystals for her, and only saw her occasionally. Finding that the effect of the quantity allowed was not as marked or soothing as formerly, and wishing to secure a speedy result, she gradually increased the dose. Soon a mild diarrhea supervened, but this did not arouse my suspicions. At this time, I should judge that she was taking about 150 grains daily. She became very nervous, and her family noticed some signs of periodical excitement, but no importance was attached to it. The dose was still further increased by her without my knowledge, and in a few days, it then being about two months from the inception of the treatment, she suddenly left the house, and, going to a river near at hand, attempted to drown herself. When rescued, she still showed a tendency to destructive outbreaks with unrestrained violence of temper. This suggested the possibility of bromic influence, and the discontinuance of the bromides at once proved the correctness of the supposition, for after that, except when once again subjected to the same treatment, did she ever exhibit any symptoms that might rise to the danger line. Dr. Draper relates the history of a case in a young man who was an epileptic, who could not take 60 grains a day for a week without becoming homicidal.

9. Bromides weaken the nervous system.

It is evident to my mind that these salts, by reason of their characteristic physiological action, must, in continued doses or large doses, depress certain organic functions and therefore be weakening to the nervous system, for in order that a sedation of the nerve centers may be secured, they diminish the action of the heart, lower the temperature, and consequently lessen the blood supply to various organs. Their field of therapy is limited, and I am skeptical if their efficacy, even in temporarily controlling the nervous explosions of the epileptic, is commensurate with the deleterious effects to the patient's general constitution. A patient addicted to the bromic habit presents a picture not easily effaced from memory, for his pallid face, dilated eyes, weak and tremulous gait, are only too patent evidence of a nervous wreck and a blighted mentality.

My object in presenting this paper at this time is to direct attention to the unusual or exceptional baleful effects of the bromides in continuous or unnecessary large doses, for, as a practitioner, I realize that our profession, and consequently the public at large, are not cognizant of them, and hence not alive to the dangers incident to their persistent use.

Too little care is often used in prescribing these drugs, and too little attention is paid to the proper regulation of the dose; circumstances, in my opinion, which are responsible for the great and increasing harmful consumption of the numerous proprietary preparations of the bromides which are now used by our people as "headache" cures, "insomnia" cures, etc., and which owe their popularity and efficacy to a varying proportion of the bromides in their composition. It is not so much our indifference, as physicians, to the best methods of prescribing the bromides for specific purposes, as our carelessness in not informing our patients of the danger of these preparations, whereby they are all but unconsciously contracting the "bromo" habit, that we are reprehensible. It is not the small and occasional dose that ultimately wrecks the constitution, but the continuous daily use, in many cases, for every nervous manifestation, as conceived and diagnosed by the layman himself without the knowledge or direction of the physician.

### BIBLIOGRAPHY.

(1) Wood's Therapeutics and Pharmacology.
(2) Archives Générales.
(3) Materia Medica and Therapeutics.
(4) University Medical Magazine, June, 1896.
(5) Journal of Inebriety, July, 1896.
(6) Traité de Therapeutique.
(7) Medical and Surgical Reporter, January, 1870.
(8) Journal of Inebriety, July, 1896.
(9) Traité Matière Médicale (4th ed.).
(10) Diseases of Nervous System.

---

### THE MORALS OF A SURGEON.

What a man does is the proof to the world of what a man is. Many good people fear that the advance of science will bring about the retrogression of morals and religion. We do not agree with them. But if they cannot accept our judgment, let them weigh well a fact like this: Mr. Jonathan Hutchinson, F.R.S., and ex-president of the Royal College of Surgeons, addressed his professional brethren assembled in annual congress the other day, and he thus spoke: "I bore with such equanimity as I could the discovery that I could not compete with my friend in the ratio of successes obtained" (in operations for ovariotomy), "and, acting on the rule of conduct that I would never keep a patient in my own hands if I believed that some one else could do what was needed with greater prospect of success, I gave up doing ovariotomies, both in public and in private, and used to transfer my patients from the London to the Samaritan Hospital." Here is a rule of conduct which has never been excelled in moral worth in any department of professional life or private behavior. A most far-reaching and truly noble rule is this of Mr. Jonathan Hutchinson's; and the fact that he announced it toward the close of his career, in the hearing of hundreds of his professional brethren, who are almost as familiar as he is himself with the conduct of his professional life, is proof that he spoke mere truth. If these are the morals of men of science may we not say of men of all professions and callings, "O si sic omnes!"—*The Hospital.*

